# Using brothel leadership to promote condom use among brothel-based female sex workers in Abuja, Nigeria: study protocol for a cluster randomized pilot trial

**DOI:** 10.1186/s40814-017-0124-0

**Published:** 2017-02-20

**Authors:** Uchenna Okafor, Rik Crutzen, Ifeanyi Okekearu, Sylvia Adebajo, Adaora Uzoh, Egbe Aneotah Awo, Chukwuemeka Chima, Ogechukwu Agwagwa, Bart van den Borne

**Affiliations:** 1 Maastricht University, Maastricht, The Netherlands; 2 Society for Family Health, Abuja, Nigeria; 3 Population Council International, Abuja, Nigeria

**Keywords:** HIV prevention, Intervention, Protocol, Female sex workers, Brothel, Condom use

## Abstract

**Background:**

The HIV prevalence among female populations involved in sex work in Nigeria has heightened interest in HIV prevention programming for this sub-population with brothel-based female sex workers (BB FSWs) having a prevalence of 27.4%, six times higher than the prevalence in the general population.

**Methods/design:**

The clusters in the randomized pilot trial will be brothels and female sex workers (FSWs) residing in the brothels will be the participants of the study. The participants will receive free condom distribution as well as HIV prevention messages on condom use and negotiation skills to increase self-efficacy in handling social and gender power plays within their environment. Twelve brothels will be randomized into experimental and control conditions with a minimum total sample size of 200 participants. Recruitment of participants will be carried out from within the brothels. The control condition will receive a standard intervention consisting of a minimum of six interactive sessions with peer educators (PE) engaging their peers through group discussions and one on one interaction using pre-designed HIV prevention messages. The experimental condition will receive the standard intervention as well as interactive sessions with the brothel leadership (chairladies and brothel managers) to facilitate consistent condom use and appropriate condom use policies, conditions, and messaging. Both interventions will be delivered over a maximum period of 16 weeks, and male and female condoms will be distributed during the intervention. Quantitative assessments will be carried out at baseline and at 16 weeks follow-up, and the pilot findings will inform feasibility of and sample size estimation for a phase III trial. The primary outcomes measured are recruitment rate attrition rate and adherence to the intervention. Consistent condom use outcomes by FSWs within the brothel with all partner types and enhanced self-efficacy for condom negotiation with all partner types will be the primary outcomes for the main study, and the feasibility of their measurement will be determined in this pilot trial.

**Discussion:**

The manuscript describes the protocol for a pilot study to determine the feasibility of a behavioral intervention to improve consistent condom use among BB FSWs. The results of this pilot will inform a larger intervention for HIV prevention for this target group in Nigeria.

**Trial registration:**

The Institutional Review Board (IRB) of the Institute of Human Virology, Nigeria; Protocol Number NHREC/10/15/2014a-026.

## Background

The HIV epidemic continues to constitute serious socioeconomic and health challenges in sub-Saharan Africa with Nigeria ranked second in the number of people living with HIV/AIDS after South Africa [1]. The Integrated Biological and Behavioral Surveillance Survey conducted by the Federal Ministry of Health in Nigeria in 2010 showed HIV prevalence of 27.4 and 21.7% among brothel-based and non-brothel-based female sex workers (FSWs), respectively [2]. FSWs have been identified as a key population at risk of HIV transmission and, directly, FSWs injecting drug users and men who have sex with men constitute about 1% of the adult population but contribute about 23% of new HIV infections [3, 4].

Several studies conducted in Nigeria and other countries around the world indicate that the rate of condom use with steady partners is significantly lower than that with clients [3–8]. Steady partners (e.g., boyfriends, husbands, fiancés, and regular paying partners) are distinct from clients and have different meanings to FSWs. They often wish to avoid rejection by steady partners in addition to the need to distinguish their work lives from their personal lives and to be trusted and loved [5, 9]. The inconsistent use of condoms with steady partners is a barrier to effective HIV prevention for FSWs and their partners that needs to be overcome [8].

Given the multifaceted nature of environmental factors influencing the sexual behavior of FSWs in Nigeria [8], an important influential group for FSWs is the brothel leadership (brothel owners, managers, chairladies, and madams). They are key power structures governing the immediate environment of the FSW, and within the brothel sex work setting, they can make access to the sex workers easier which can be useful in the communication of key messages and information. These individuals have been shown in some studies to effectively influence the sexual behavior of FSWs whom they have direct influence over [10, 11].

We conducted two different studies as formative research for the development of an intervention aimed at increasing condom use among FSWs [12]. A review of literature which showed the limited incorporation and measurement of the effect of using brothel leadership in condom promotion interventions (Okafor et al., submitted-a) and an analysis of secondary data from the Integrated Biological and Behavioral Surveillance Survey (Okafor et al., submitted-b) revealing the vulnerability of the young sub-population of both brothel-based female sex workers (BB FSWs) and non-brothel-based FSWs with the BB FSWs showed comparatively higher vulnerability, HIV prevalence, and risk profile.

This study aims to use a cluster randomized pilot trial approach to gain more insight into the potential of an intervention using brothel leadership to improve consistent condom use by FSWs residing in brothels. The objectives of this pilot study are to gain insight into:

1. The feasibility of implementing the intervention within brothels
2. The attrition rate for FSWs residing within the brothels
3. The potential effect of the intervention on consistent condom use for FSWs within the brothel with all partner types in order to inform a sample size estimation for an adequately powered phase III trial

## Methods/design

### Development process

For the development of the intervention and to facilitate the design of appropriate activities [12] for all levels of the intervention (individual, interpersonal, and organizational levels), the intervention mapping approach was used [13]. A systematic review of literature and analysis of secondary data on FSWs provided information on the vulnerability of BB FSWs and the availability of limited information on the effect outcomes related to the inclusion of brothel leadership in condom use intervention for FSWs in Nigeria. A planning group was set up consisting of HIV prevention specialists, FSWs, and brothel owners. All members of the planning group were engaged directly, and several discussion sessions were conducted to obtain more insights into the target population; FSW community, existing programs, and strategies successfully deployed for HIV prevention interventions. The HIV specialist panel consisted of seven experts; three females and four males. Five focus group discussions were conducted with FSWs residing in brothels within Abuja, Nigeria. An analysis of program details within an existing HIV prevention program; the Strengthening HIV Prevention Services for Most at Risk Populations (SHiPs for MARPS) project was also carried out. The SHiPs for MARPs project is a HIV prevention project in Nigeria funded by USAID and targeted at most at risk populations: FSWs, men who have sex with men, and injecting drug users. The behavioral components of the project uses peer-mediated behavioral approaches to promote consistent condom use among BB FSWs, and this component of the project served as the standard intervention for the pilot study.

We brought together eight selected brothel leaders and presented components of the proposed intervention to them to get their insights on its suitability for the brothels. Using all the information obtained, we developed an intervention to improve consistent condom use by BB FSWs.

### Theoretical model

The theoretical model underlying the intervention process is the Social Cognitive Theory (SCT). The SCT postulates that people and their environment interact continuously and the environment shapes, maintains, and constrains behavior, but people are not passive in the process, as they can create and change their environments [14]. The SCT focuses on the individual, community, and organizational levels that influence the individuals’ power over their behavior. The individual level is characterized by awareness and motivational determinants (i.e., knowledge, awareness, risk perception, and self-efficacy). Self-efficacy is crucial in changing health behaviors and is proximal to preparing and deciding to act. The interpersonal/community level includes peer modeling for observational learning to enhance emulation of desired behavior by watching the actions and consequences for others who are credible role models of the targeted behavior. The brothel owners, managers, and chairladies are crucial agents at the organizational level to facilitate the existence of appropriate conditions conducive for improved self-efficacy and adoption of the required behavior by providing appropriate support and materials. Their involvement is useful in facilitating the consistent use of condoms with clients and steady sexual partners of FSWs.

### Study design and setting

The study will be conducted in Abuja, Nigeria, and intervention components will be evaluated using a cluster randomized pilot trial approach, comparing a standard intervention (i.e., usual care) to a standard intervention in combination with the brothel leadership intervention. Randomization of the study participants at the brothel level will be carried out to limit contamination between the two intervention groups.

Clusters will be brothels with a minimum of five FSWs and presence of FSWs aged from 15–24 years of age. The criteria for inclusion into the study include females who undertake sexual activities with men in return for money or benefits, who reside in brothels, who provide verbal informed consent, as well as brothels with chairladies who provide consent for the study.

The structure of the study, key messages, monitoring tools, and training slides were developed with information obtained from the formative studies and several meetings with the HIV prevention specialists. The messages and interactions for the intervention will focus on improving consistent condom use and condom negotiation self-efficacy of FSWs residing in brothels. The training of the brothel leaders will be carried out prior to the intervention, and peer educators from the pool of trained peer educators within the existing SHiPs for MARPs project will be used. The activities for both the standard and the brothel leadership intervention groups will be implemented for 16 weeks and will include interactive sessions with the peer educators engaging their peers through group discussions and/or one on one interaction using pre-designed HIV prevention messages. Male and female condoms will be distributed for free during the intervention period. Various studies have shown that the FSW population is a migrant population with high mobility [15, 16], and this poses a challenge during implementation for this target group. To minimize this and facilitate follow-up of the girls within the intervention, the intervention will be carried out for 16 weeks and messages will be reinforced using information and communication materials (i.e., posters and leaflets with condom use messages).

### Sample size

Based on recent experience in local brothels, we anticipate that there will be between 5 and 50 FSWs in each brothel. Since this is a pilot study with the aim of providing information on the feasibility, attrition rates, and sample size estimates for a larger pilot trial, we aim to recruit 12 brothels. The formula provided by Viechtbauer et al. [17] for the calculation of sample size required for a pilot was applied in the determination of the sample size using a confidence interval of 95% and a 5% probability of existence of a problem. This resulted in a required sample size of 59 to enable the detection of feasibility problems. Due to the high attrition rate often reported in studies with FSWs and the lack of previous knowledge regarding clustering in brothels, it is unlikely that we will have all the recruited participants at follow-up; thus, we aim to recruit 200 participants to account for both aspects.

### Allocation strategy

**Fig. 1:**
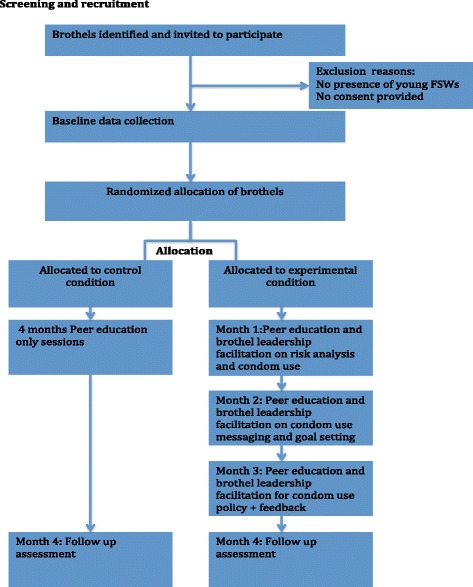
Intervention flow diagram

The brothel is the unit of randomization, and after baseline data collection, the brothels will be ordered alphabetically by name of brothel. Each brothel will be allocated a number sequence for random assignment to the experimental or the control conditions. To ensure balance in brothel size in both study groups, stratification by brothel size will be carried out to ensure mix of both large and small brothels in both groups. Allocation of brothels will be done by an independent member of the research team blind to the characteristics of the brothels, and this individual will inform the pilot team on the status of each brothel. All FSWs within each selected brothel who give consent for the study will be eligible for enrollment in the study (Fig. 1).

### Standard intervention

The standard intervention is a behavioral intervention using the combination prevention approach consisting of peer mediated sessions on risks and vulnerabilities, condom use promotion, and condom distribution. The peer sessions will be a 1-h group discussion conducted within the brothel, and each peer will be exposed to a minimum of six sessions during the intervention period. The peer sessions will be carried out within the brothel by selected FSWs trained as peer educators (PEs) by the project staff. Topics to be discussed during the group sessions include life skills, sexuality, risk reduction, money and conflict management, and consistent condom use. Project facilitators will be assigned to each brothel, and the FSWs will be provided with hygiene kits, weekly distribution of female and male condoms, and information on referral services available at drop in centers, i.e., HIV testing and counseling (HTC) and syndromic management of sexually transmitted infections (STIs).

### Brothel leadership intervention

In addition to the activities described in the standard intervention, the pilot intervention will include using the brothel leadership (brothel managers, chairladies, and owners) to deliver condom use messages to the FSWs and to promote a climate within the brothels for communication, condom negotiation, and condom use with all sexual partners within the brothel. The messages and interactions for the intervention will focus on improving condom use and condom negotiation self-efficacy of the FSWs

The messages will be differentiated to target the psychosocial issues surrounding condom use with the different partner types: clients and steady partners. These brothel leaders will be given a one-day workshop on facilitating the promotion of consistent condom use among the FSWs in their brothels using active learning and facilitation methods to encourage learning, identify barriers, and facilitate consistent condom use. The workshop will be practice-oriented and provide opportunities to rehearse session delivery and condom demonstrations. The workshop will be facilitated by HIV prevention experts from the SHiPs for MARPs project with the modules covering the following topics: HIV knowledge, risk assessment, and common misconceptions; discussions on benefits of condom use to the establishment and FSWs; group work on roles of gatekeepers for the improvement of condom use in brothels; role plays on condom demonstration and condom use negotiation. At the end of the workshop, participants will be given leaflets showing the activities to be carried out by each brothel leader for the intervention. Project staff will also be assigned to support each brothel during the intervention period. After 8 weeks, a refresher workshop will be conducted for the brothel leaders to discuss implementation milestones and issues encountered.

Brothel leaders are authority figures within the brothels and are able to regulate and oversee the activities of the peer educators and the FSWs within their brothels ensuring peer sessions are carried out and facilitating condom promotion activities within the brothel. Each leader will ensure that a minimum of six group sessions is carried out within the brothel. During these sessions, discussions on FSW’s risk profile, condom demonstrations, free distribution of male and female condoms, as well as the setting of rules and regulations to facilitate consistent condom use within the brothel will be carried out. New entrants within the brothel will also be taken under the wings of the brothel leaders to foster adherence by introducing them to the consistent condom use policy.

The group meetings by the brothel leadership will include all FSWs within the brothel and serve as platforms to address barriers to condom use and reinforce condom use through condom demonstrations. Posters and leaflets with condom use messages will be distributed, and the chairladies and managers will be assisted at the end of the intervention to stock condoms for continued availability of condoms within the brothel.

### Consent process

The verbal consent process will be used to obtain consent for participation in the study as a result of the low literacy level of the FSWs. The interviewer will read the consent script to the participant. Once the consent is read, the participant will be given time to ask questions. The participant will verbally agree to participate, and the interviewer will sign the consent form indicating this verbal consent.

### Measurements

The primary outcomes of this study are determining the feasibility of working with brothel leadership in deploying condom use messages within brothels (measurement, the number of brothels consenting to the intervention); the attrition rate of FSWs residing within the brothels (measurement, the proportion of FSWs and brothels completing the intervention in the intervention monitoring database); and the adherence to intervention (measurement, number of sessions attended by each FSW). The secondary outcomes are to determine the feasibility of measuring the primary outcomes for the main trial (measurement, FSWs self-reported consistent condom use in the baseline and follow-up assessments) and enhancing self-efficacy in condom use negotiation with all clients and partners of FSWs (measurement, FSWs self-reported self-efficacy regarding the ability to negotiate condom use with different partner types in the baseline and follow-up assessments).

For the process outcomes, participants will be asked several questions at the end of the 16-week follow-up period using interviewer-administered semi-structured questionnaires. The process evaluation will measure the acceptability, suitability, content, and mode of delivery (measurement, self-reported adequacy of the duration, content, and delivery method of the intervention in the follow-up assessment).

### Statistical analysis and data management

The overall recruitment and attrition rates of the brothels and participants for the intervention arm will be described. The Intra Cluster Correlation (ICC) and the effect size will be generated in addition to the attrition rates to enable sample size estimation required for a phase III trial.

As the FSWs are nested in brothels, we will use a multilevel regression approach with three levels to assess the potential effects and viability of the intervention on condom use behavior. The first level consists of the repeated measures within the participants (baseline and follow-up measurements), the second level are the FSWs, and the third level are the brothels, where the FSWs are nested. Multilevel logistic regression will be used to analyze the effects on condom use separately for steady partners and clients. The multilevel regression analysis was chosen because this allows for data missing at random, which is less strict than the requirement of data missing completely at random [18]. Each interviewed participant will be given a code, and the questionnaires and data sets will be stored in a password-protected computer and only researchers will have access to the data.

### Dissemination of findings

The findings from the study will be published as a journal paper and presented at an international conference.

## Discussion

This paper presents the protocol that describes a pilot study to assess the feasibility and potential effect of an intervention aimed at increasing consistent condom use and improving condom negotiation skills of BB FSWs in Abuja, Nigeria. Should the current study demonstrate feasibility, outcomes will be used to design and inform a phase III pilot cluster randomized control trial (CRCT). The aim of the phase III pilot will be to further test the effectiveness of the behavioral intervention in improving consistent use of condoms by BB FSWs in Nigeria.

The mobility of sex workers is one of the challenges faced when programming for this target group [16]. To minimize this, the intervention is designed such that the core messages and activities are delivered within a short time span, thereby enabling most of the FSWs to benefit from it before they may need to change their location.

Formative research was conducted in collaboration with the target group and the brothel leaderships for the development of the interventions; this identified important issues for the development and relevance of the intervention. Involving the brothel leaderships in the development process initially led to some resistance due to their perceived notion that the FSWs will become distracted and that they will lose the girls within their brothels. Assurance of the benefits of the intervention on the health of the FSWs and the reputation of the brothel establishment as a whole was provided. The brothel leaders were enthusiastic about the intervention and provided the needed support, without which, it will be impossible to implement the intervention.

### Trial status

The pilot phase is underway; recruitment and implementation for the next phase of the intervention is due to be completed by May 2017. The first brothel for this trial was randomized in July 2015.

## Abbreviations

BBFSW: Brothel-based female sex workers
CRCT: Cluster randomized control trial
FCT: Federal Capital Territory
FSW: Female sex workers
HIV: Human immunodeficiency virus
HTC: HIV testing and counseling
IRB: Institutional review board
PE: Peer educators
SCT: Social cognitive theory
SHiPs for MARPs: Strengthening HIV Prevention Services for Most at Risk Populations
STI: Sexually transmitted infections
